# Adaptive coding in the human brain: Distinct object features are encoded by overlapping voxels in frontoparietal cortex

**DOI:** 10.1016/j.cortex.2018.07.006

**Published:** 2018-07-25

**Authors:** Jade B. Jackson, Alexandra Woolgar

**Affiliations:** a King's College London, Institute of Psychiatry, Psychology and Neuroscience, Department of Neuroimaging, London, United Kingdom; b Perception in Action Research Centre and Department of Cognitive Science, Macquarie University, and ARC Centre of Excellence in Cognition and its Disorders, Macquarie University, Sydney, Australia; c MRC Cognition and Brain Sciences Unit, University of Cambridge, Cambridge, United Kingdom

**Keywords:** MVPA, fMRI, Adaptive coding, Voxel re-use

## Abstract

Our ability to flexibly switch between different tasks is a key component of cognitive control. Non-human primate (NHP) studies (e.g., Freedman, Riesenhuber, Poggio, & Miller, 2001) have shown that prefrontal neurons are re-used across tasks, re-configuring their responses to code currently relevant information. In a similar vein, in the human brain, the “multiple demand” (MD) system is suggested to exert control by adjusting its responses, selectively processing information in line with our current goals (Duncan, 2010). However, whether the *same* or *different* resources (underlying neural populations) in the human brain are recruited to solve different tasks remains elusive. In the present study, we aimed to bridge the gap between the NHP and human literature by examining human functional imaging data at an intermediate level of resolution: quantifying the extent to which single voxels contributed to multiple neural codes. Participants alternated between two tasks requiring the selection of feature information from two distinct sets of objects. We examined whether neural codes for the relevant stimulus features in the two different tasks depended on the same or different voxels. In line with the electrophysiological literature, MD voxels were more likely to contribute to multiple neural codes than we predicted based on permutation tests. Comparatively, in the visual system the neural codes depended on distinct sets of voxels. Our data emphasise the flexibility of the MD regions to re-configure their responses and adaptively code relevant information across different tasks.

## 1 Introduction

To function successfully, we need a cognitive system that can select what is relevant for our behaviour and ignore distraction. Moreover, this system needs to constantly update the way it responds, to meet the requirements of our current goals. However, we do yet not fully understand how the human brain is able to swiftly adjust its processing priorities in response to our constantly updated goals.

Different mechanisms may underlie our ability to do this efficiently. For example, performance across different tasks could rely on distinct specialised neural resources. The rule abstraction model of prefrontal function (Badre, 2008; Badre & D'Esposito, 2009) suggests a rostrocaudal gradient where distinct regions are recruited according to differing task demands. An alternative possibility is that neurons may flexibly code many different types of task information. The adaptive coding hypothesis (ACH), proposes that context-specific parameters shape the tuning profile of higher cortical neurons (Duncan, 2001, 2010). Rather than being tuned to specific features in the environment, these neurons are proposed to have highly adaptable response properties, coding information according to what is currently relevant for behaviour.

Evidence for ‘adaptive coding’ stems primarily from NHPs. Prefrontal neurons flexibly encode the behavioural significance of visual stimuli (i.e., coding is dependent on task parameters), regardless of their physical properties (e.g., Cromer, Roy, & Miller, 2010; Freedman, Riesenhuber, Poggio, & Miller, 2001; Roy, Riesenhuber, Poggio, & Miller, 2010). For example, in Cromer and colleagues' (2010) study, NHPs classified stimuli according to an arbitrary category boundary. Individual prefrontal neurons displayed tuning profiles that were aligned with the task-relevant decision space. When NHPs were required to classify a second group of stimuli according to a new decision boundary, the firing rate of 44% of these neurons changed to reflect the new task. These data emphasise that the response of prefrontal neurons changes flexibly to reflect the currently relevant information. In this way, single neurons are re-used in multiple neural codes.

In the human brain, the MD regions are considered candidate regions for adaptive coding (e.g., Duncan, 2010). They are defined as regions that are active for a wide range of task demands (Duncan & Owen, 2000; Fedorenko, Duncan, & Kanwisher, 2013) and consist of cortex in and around the inferior frontal sulcus (IFS), anterior insula/frontal operculum (AI/FO), pre-supplementary motor area and dorsal anterior cingulate (pre-SMA/ACC), and intraparietal sulcus (IPS). Using multi-voxel pattern analysis (MVPA), these regions have been shown to code a range of task features (e.g., Bode & Haynes, 2009; Erez & Duncan, 2015; Harel, Kravitz, & Baker, 2014; Haynes et al., 2007; Nee & Brown, 2012; Reverberi, Gorgen, & Haynes, 2011; Stiers, Mennes, & Sunaert, 2010; Woolgar, Jackson, & Duncan, 2016; Woolgar, Williams, & Rich, 2015) and these codes adjust when task demands vary (Li, Ostwald, Giese, & Kourtzi, 2007; Woolgar, Hampshire, Thompson, & Duncan, 2011; Woolgar, Afshar, Williams, & Rich, 2015; Woolgar, Williams, et al., 2015). Moreover, we have demonstrated that MD codes emphasise different aspects of visual objects as required by the current task (Jackson, Rich, Williams, & Woolgar, 2017). At the level of whole regions at least, the MD regions appear to code different task information according to what is currently relevant for behaviour.

The ACH and NHP studies (e.g., Cromer et al., 2010) consider the responses of individual neurons. However, neuroimaging studies of MD function have only examined whole region responses. Do the human results reflect adaptive coding of individual neurons, like that of NHPs, or do they simply reflect the responses of multiple independent specialised neural populations that lie within the MD regions? Here, we intended to bridge the gap between the human and NHP data by considering an intermediate level of resolution: the extent to which different neural codes load on the same individual voxels. We refer to this as “voxel reuse”, an index of the extent to which the same voxels contribute maximally to the multivoxel codes for two distinct task features. To examine this, we first used MVPA to extract the multi-voxel codes that distinguished the task-relevant features of objects in two separate tasks. Then, we developed a method to measure the extent to which the *same* voxels in the MD regions were re-used in the two codes (coding relevant information for different groups of objects). We compared the extent of voxel re-use against the chance-level derived from permuting the data. At this intermediate level of resolution, single voxels could of course still sample multiple overlapping neural populations, so we cannot draw conclusions at the single neuron level. However, we reasoned that if the two codes depended on independent voxel populations within the MD regions, this would provide evidence against the ACH. We predicted that the same MD voxels would contribute to coding of relevant information across different tasks, whilst voxels in more specialised brain regions (early visual cortex) would not.

## 2 Materials and methods

### 2.1 Participants

Twenty-six participants (17 females; mean age = 23.9 years, SD = 4.56) were recruited from the Macquarie University Psychology Participant Pool. All participants were right-handed with normal or corrected-to-normal vision and no history of neurological or psychiatric disorder. Participants gave written informed consent and received $50. The experiment was approved by the human research ethics committee of Macquarie University (Sydney, Australia).

### 2.2 Stimuli

**Fig. 1:**
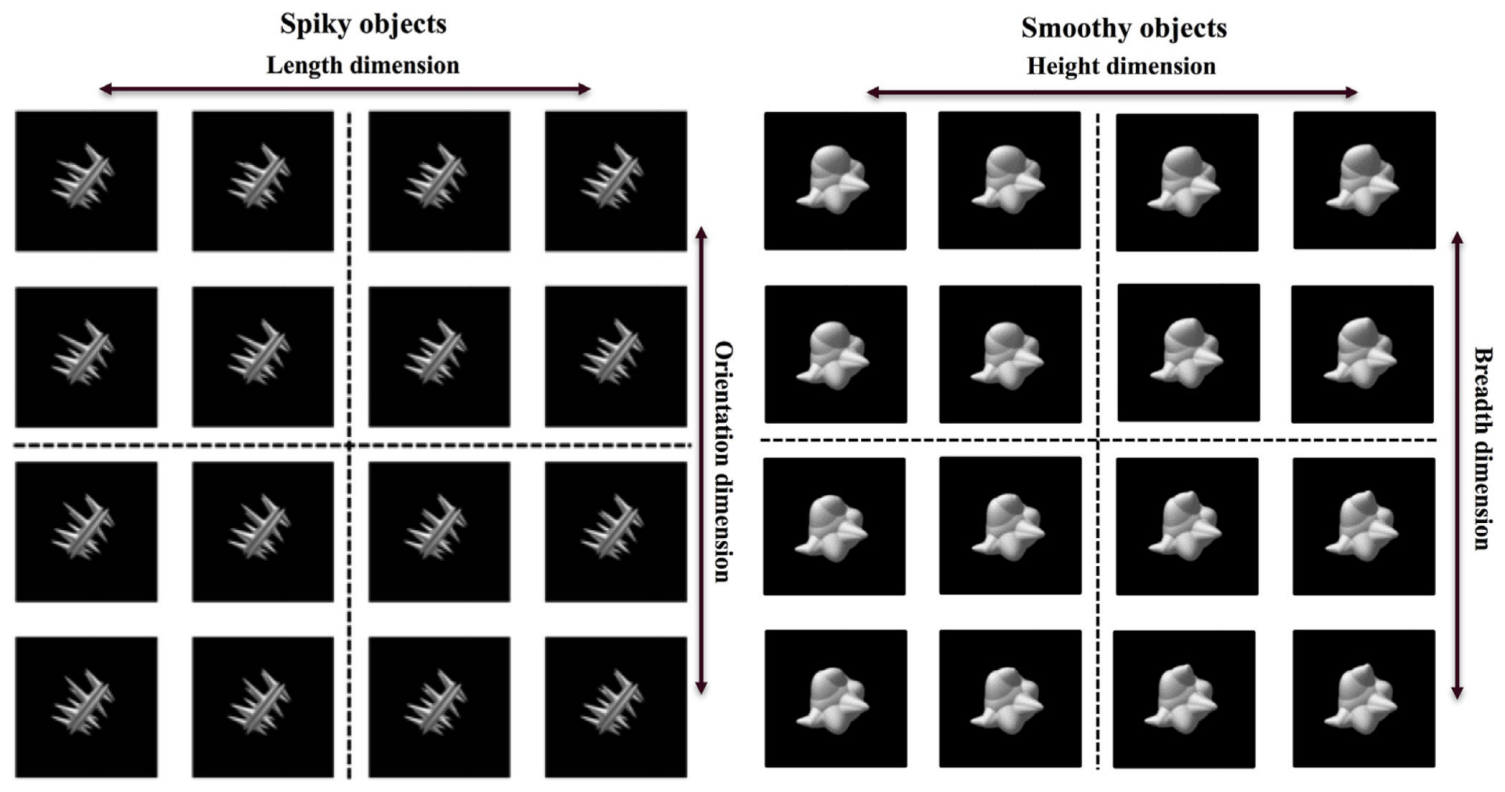
Stimulus set. The stimulus set consisted of 32 objects total. The visual angle of the spiky object's length along its main axis was 8.07° and for the smoothy objects it was 8.56° . One “spike” of the spiky objects varied along two dimensions (its length and orientation) and one “spheroid” of the smoothy objects also varied along two dimensions (its breadth and height). Participants categorised the spiky objects according to the orientation dimension; the length dimension was always irrelevant. For the second task, participants categorised the smoothy objects according to breadth dimension; the height dimension was always irrelevant. Stimuli were presented at central fixation on a screen and viewed through a mirror mounted on the head coil in the scanner.

The stimulus set consisted of abstract novel “spiky” and “smoothy” objects (Fig. 1) created using custom MatLab scripts (Op de Beeck, Baker, DiCarlo, & Kanwisher, 2006). One “spike” of the spiky objects varied along two dimensions (length/orientation) and one “spheroid” of the smoothy objects also varied along two dimensions (breadth/height). The design followed our previous work (Jackson et al., 2017), but following a hint in the NHP literature that neural re-use may be larger for dissimilar object tasks (Cromer et al., 2010), we chose to test voxel re-use across two distinct sets of objects.

Participants performed two tasks. In one task, participants discriminated between the spiky objects based on the orientation dimension (rotated clockwise *vs*. anti-clockwise spikes). For the second task, subjects discriminated the smoothy objects based on the breadth dimension (wide *vs*. narrow spheroid). Stimulus presentation was controlled by a PC running the Psychophysics Toolbox-3 package (Brainard, 1997) in Matlab (Mathworks).

### 2.3 Procedure

**Fig. 2:**
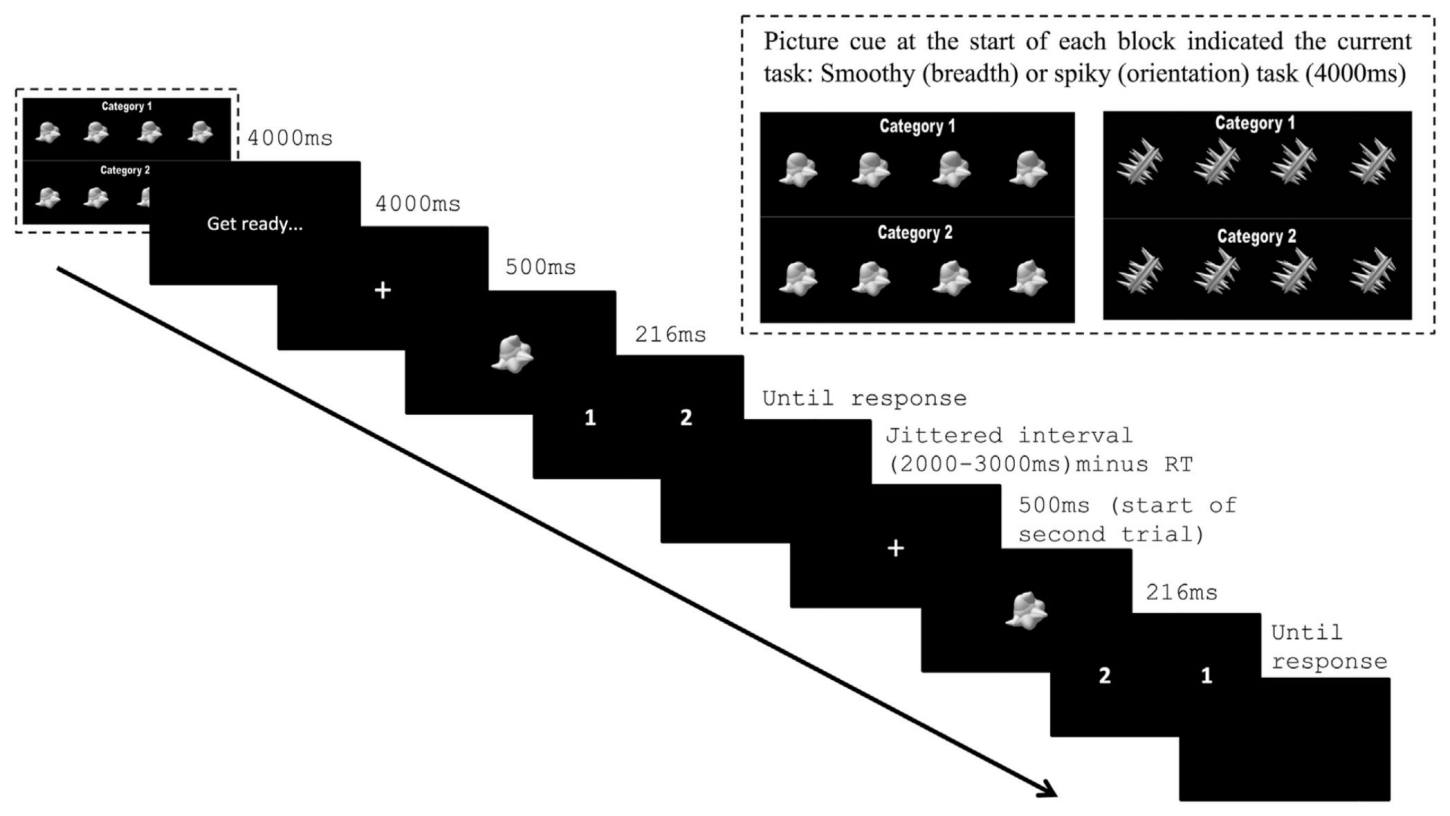
Stimulus categorisation task. A picture cue at the start of each block indicated the current task: Breadth (smoothy task) or orientation (spiky task). The inset shows cue display for both the orientation and breadth task. On each trial a fixation cross was presented for 500 msec followed by an object for 216 msec. Finally, a response mapping screen appeared which indicated the appropriate response button. The response mapping screen randomly assigned category 1 and 2 decisions to either the left or right response button, operated by the index or middle finger of the participant's right hand. The response mapping screen was visible until a button-press was made or until the jittered time interval timed out (2000–3000 msec). If a response was made before the end of the inter-trial interval, a blank black screen was shown for the remainder of the trial time. In the example shown here, the current task is breadth (smoothy). For the first trial, the stimulus is category 2 on the breadth dimension and therefore the correct response was the right-button.

Prior to entering the scanner participants practised the task and stimuli were titrated to match the difficulty between the tasks (See S1). Participants then completed 4 acquisition runs (8.09 min each) consisting of 4 blocks of 128 trials. At the start of each block, a cue (4000 msec) indicated the current task (orientation of the spikes, breadth of the spheroids; block order counterbalanced within and across participants), see Fig. 2. The cue also indicated which attribute was category 1 and 2 (e.g., whether rotated clockwise/anti-clockwise spikes were category 1 or 2; counterbalanced across participants). On each trial, participants saw a white central cross (500 msec) followed by an object (216 msec) that they categorised according to the current task. Finally, participants saw a response mapping screen that indicated the category-to-button response mapping on this trial. At the end of each block participants saw feedback (% correct; 6000 msec) then a blank screen (4000 msec). At the end of each run, an additional blank black screen was shown for 4000 msec.

Participants also completed a localiser task to functionally identify the lateral occipital complex (LOC) as a region-of-interest (ROI). Participants viewed centrally presented intact and scrambled versions of black and white natural objects in 16.8s blocks of 16 trials (1100 msec/trial), whilst attending to a central fixation cross. Participants pressed a button each time the fixation cross changed colour. There were 21 blocks consisting of alternating blocks of whole objects, scrambled objects, and rest. The localiser took 6.25 min.

### 2.4 Data acquisition

FMRI data were collected using a 3T Siemens Verio Magnetic Resonance Imaging scanner at Macquarie University Hospital. We used a sequential descending T2*- weighted echo planar imaging (EPI) acquisition sequence: acquisition time 2000 msec; echo time 30 msec; 34 oblique axial slices collected in descending order; slice thickness 3.0 mm; .70 mm interslice gap; in plane resolution 3.0 × 3.0 mm; matrix 64 × 64; field of view 210 mm; flip angle 78°. We also acquired T1-weighted MPRAGE structural images (slice thickness 1.0 mm, resolution 1.0 × 1.0 mm).

### 2.5 Preprocessing

MRI data were preprocessed using SPM 5 and SPM 12 (Wellcome Department of Imaging Neuroscience, www.fil.ion.ucl.ac.uk/spm) in Matlab 2011b. Functional MRI data were converted from DICOM to NIFTII format, spatially realigned to the first functional scan and slice timing corrected. EPIs from the main experiment were smoothed slightly (4 mm FWHM Gaussian kernel) to improve signal-to-noise ratio, as in our previous work (Woolgar, Afshar, et al., 2015; Woolgar, Williams, et al., 2015; Jackson et al., 2017). Localiser EPIs were also smoothed (8 mm FWHM Gaussian kernel) and in all cases the data were high pass filtered (128s). Structural scans were co-registered to the mean EPI and normalised, using the segment and normalise routine of SPM5, to derive the normalisation parameters needed for ROI definition and to normalise individual participant searchlight classification maps.

### 2.6 Regions of interest

MD ROIs were defined using co-ordinates from a previous review of activity associated with a diverse set of cognitive demands (Duncan & Owen, 2000) using the kernel method described in Cusack, Mitchell, and Duncan (2010) as in our previous work (Woolgar, Hampshire, et al., 2011; Woolgar, Thompson, Bor, & Duncan, 2011; Woolgar, Williams, et al., 2015; Jackson et al., 2017).

Left and right Brodmann area 17 (BA 17) were derived from the Brodmann template provided with MRIcro (Rorden & Brett, 2000). Left and right inferior temporal cortex (IT) were derived from the Harvard–Oxford Cortical Structural Atlas provided with FSL (Jenkinson, Beckmann, Behrens, Woolrich, & Smith, 2012). MD, BA17 and IT ROIs were deformed for each participant by applying the inverse of the participant's normalisation parameters. This allowed analyses to be carried out using native space EPI data.

We defined LOC for each participant, based on the functional localiser scan, as the lateral occipital area that responded more strongly to pictures of natural/madeemade objects than to scrambled versions of the same objects. We used the standard multiple regression approach of SPM to estimate values pertaining to the whole and scrambled object conditions. Blocks were modelled using a box car function lasting 16s convolved with the hemodynamic response of SPM. The run mean was included in the model as a covariate of no interest. Whole-brain mass univariate analyses (paired *t*-tests) compared voxelwise BOLD response in the two conditions (whole objects–scrambled objects). The resulting map was thresholded such that there was at least one cluster with a minimum size of 20 voxels. We selected one left and one right cluster of activation close to anatomical LOC coordinates from previous studies (Grill-Spector, Kushnir, Hendler, & Malach, 2000; Grill-Spector et al., 1999).

### 2.7 First-level model

To obtain activation patterns for MVPA, we estimated a General Linear Model (GLM). We estimated the responses to the relevant and irrelevant features of the two sets of stimuli. For spiky objects, the relevant feature was the orientation of the spike (rotated clockwise/anti-clockwise) and the irrelevant feature was the length of the spike (short/long). For smoothies, the relevant feature was the breadth of the spheroid (wide/narrow) and the irrelevant feature was the height of the spheroid (tall/short). Every trial contributed to the estimation of two beta values; the relevant and the irrelevant feature. Trials were modelled as events of zero duration at stimulus onset convolved with the hemodynamic response of SPM5. We estimated the response for each feature (spikies; *clockwise*/*anticlockwise* and *short*/*long*, smoothies; *wide*/*narrow* and *tall*/*short*) in each block separately. The run means were included in the model as covariates of no interest. Error trials were excluded.

### 2.8 MVPA

Our aim was to investigate whether MD voxels contribute to multiple codes for relevant stimulus information in distinct groups of objects. We first established the patterns used to code for relevant information in each task, and tested the reliability of these patterns, prior to testing whether the *same* voxels were used in these codes.

#### 2.8.1 Decoding task information

We used a standard cross-generalisation MVPA approach to test the reliability of multi-voxel codes for relevant and irrelevant features of the two sets of stimuli using The Decoding Toolbox (Hebart, Görgen, & Haynes, 2015). For each participant and ROI, we trained a linear support vector machine (lSVM) to decode the relevant (clockwise or anticlockwise for spikies, and wide or narrow for smoothies) and irrelevant (short or long for spikies, and tall or thin for smoothies) stimulus features for both groups of objects (see S2. for further details). We predicted, based on our previous work (Jackson et al., 2017) that the MD network would show significant, and preferential, coding of task relevant information.

To identify any further regions showing coding of task-relevant or irrelevant information, we also performed an exploratory analysis in which we carried out classification across the whole brain using a roaming searchlight (Kriegeskorte, Goebel, & Bandettini, 2006). For each participant, data were extracted from a spherical ROI (radius 5 mm) centred in turn on each voxel in the brain. A lSVM was trained and tested using data from each sphere, and the classification accuracy value for that sphere was assigned to the central voxel. This yielded whole-brain accuracy maps for each individual. Accuracy maps were normalised and smoothed using an 8 mm FWHM Gaussian kernel for group-level analysis (one-sample *t*-test at each voxel). The results were thresholded at *p* < .001 with an extent threshold of 20 voxels. All coordinates are given in MNI152 space (McConnell Brain Imaging Centre, Montreal Neurological Institute).

#### 2.8.2 Decoding the categorical level of the decision

We conducted an additional analysis to explore whether the decision that was made by the participants was represented at the level of the stimulus (e.g., short/tall) or at the level of the category number (category 1/category 2). For this, we trained the classifier on data representing the category number decisions in one task (Breadth task; category 1/category 2) and tested on the category number decisions in the other task (Height task; category 1/category 2), and vice versa.

#### 2.8.3 Overlapping multi-voxel codes for relevant information

We developed an extension of MVPA to extract the voxels contributing the most signal to our multi-voxel codes, and to interrogate whether these voxels were the *same* voxels across multiple codes.

First, we identified the voxels that contributed the most signal to the stimulus discriminations using a transformation of the classifier weights (Haufe et al., 2014). For each participant and ROI, we trained a linear support vector machine classifier using all the data (8 blocks) in each task separately (e.g., clockwise *vs* anti-clockwise in the 8 spiky blocks). From this we extracted the weight assigned to each voxel by the classifier, and transformed it to an index of discriminatory signal strength by multiplying the classifier weights by the covariance in the data (Haufe et al., 2014). This transformation is necessary to recover the extent to which each voxel contributed signal to each multivoxel pattern. Akin to transforming the backward model (the multivariate classifier, which attempts to extract neural information from the fMRI data) to a forward model (which would specify the fMRI data given the neural information) the transformed weights are neurophysiologically interpretable, whereas the raw weights may, for example, be high for voxels that give a good estimate of covarying noise, and are therefore statistically independent of the neural signal of interest (Haufe et al., 2014).

We then calculated the voxel re-use index between the top 10% of voxels contributing the most signal to our two task-relevant codes. To do so we identified the voxels with highest (top 10%) transformed weights for orientation coding in the spiky blocks and the top 10% of voxels contributing the most signal to breadth coding in the smoothy task blocks, and asked how many of these were the same voxels. We expressed this value as a proportion. For example, if 40 out of the 200 voxels in an ROI that contributed the highest signal to the discrimination of orientation were also amongst the 200 voxels that contributed the highest signal to the discrimination of breadth, then the proportion of overlap (voxel re-use) was 40/200 = .2 (20%). We repeated this procedure for every participant, in each ROI separately.

Finally, we used a two-step permutation test (Stelzer, Chen, & Turner, 2013) to test whether the extent of voxel reuse in our data exceeds the extent expected by chance. For this, we trained a classifier on permuted condition labels and calculated voxel re-use. Next, we built a group level null distribution to calculate the probability of observing the actual voxel re-use value given the group null distribution (refer to S3. for further details). This approach accounts for within-subject factors such as vasculature that could lead to certain voxels having higher classification weights in multiple discriminations for uninteresting reasons.

This measure of voxel re-use is only meaningful in regions where patterns of activation reliably discriminated between the stimuli in the first place, so for our main analysis, we only calculated voxel re-use in conditions where information coding was above chance in the previous analysis. However, as a sanity check, we checked whether voxel re-use was at chance when information coding was at chance, and compared the proportion of voxel re-use between the task-relevant and task-irrelevant conditions in the MD network.

## 3 Results

### 3.1 Behavioural results

Prior to scanning, the stimulus set was titrated to match reaction times between the two tasks for each participant separately (assessed with Bayes factor analysis for each participant separately, using a threshold of BF < 1, all actual BF_10_ < .76) (Dienes, 2011; Love et al., 2015). In the scanning session, participants performed with a high degree of accuracy (94.2%, SD = 7.1). There were no differences in accuracy between the two conditions for any participant individually (all BF_10_ < .89). The average reaction time from stimulus onset in the scanner was 690 msec (SD = 121 msec). Reaction time data from the scanning session was were not analysed further as the response mapping screen prevented an immediate response following stimulus onset.

### 3.2 Decoding of relevant and irrelevant stimulus features

#### 3.2.1 MD regions

**Fig. 3:**
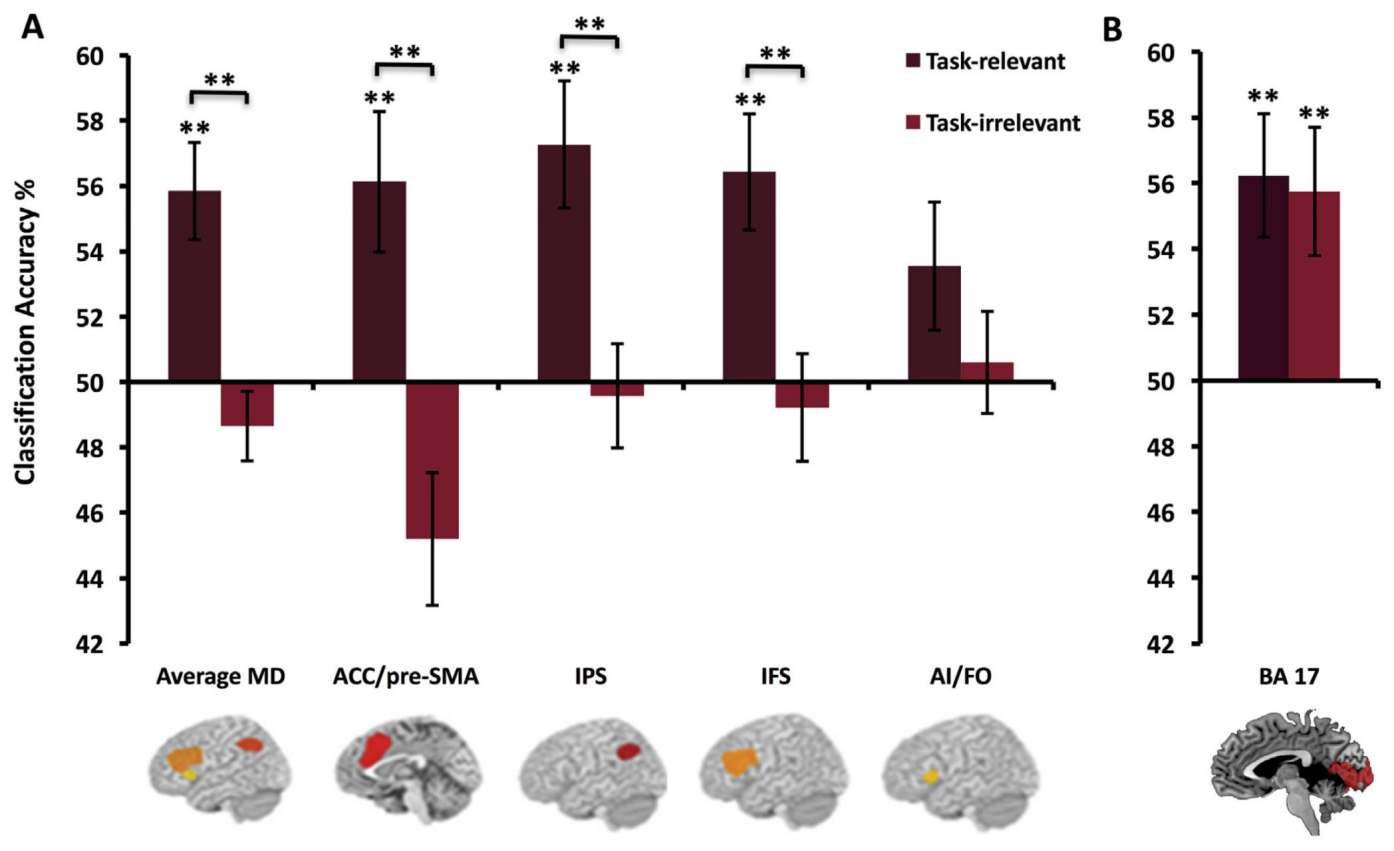
Decoding in MD network (A) and visual cortices (B). Coding of task-relevant and irrelevant stimulus distinctions in MD regions (A) and BA 17 (B). Error bars indicate standard error. Significance markings for individual bars indicate whether coding was significantly greater that chance in each condition separately (one-sample *t* test against chance, 50%), significance marking between bars indicate where coding was significantly greater for relevant compared to irrelevant distinctions (main effect of relevancy/paired *t*-test). **p < .01, alpha for individual MD regions corrected for four comparisons using Bonferroni correction (alpha level = .0125). The MD regions coded task-relevant feature distinctions more strongly than the task-irrelevant distinctions. Coding across average MD = 55.8%, *p* < .001. Relevant stimulus distinctions were coded in 3 MD ROIs; ACC/pre-SMA, MA 56.1%, *p* < .001; IPS, MA 57.3%, *p* < .001; IFS, MA 56.4%, *p* < .001, with a trend in AI/FO that did not reach our Bonferroni corrected significance level (MA 53.6%, *p* = .04). There was no coding of irrelevant feature information in any of the MD regions. An ANOVA on BA 17 classification results showed no significant main effects or interactions indicating that coding in this region was not modulated by behavioural relevance.

We predicted that the MD regions would prioritise coding of task-relevant features over task-irrelevant features. As can be seen in Fig. 3 (left panel), this was the case. A three-way ANOVA with factors *relevancy*, *region* and *object* revealed a main effect of *relevancy* (F(1,25) = 14.5, *p* = .001), corresponding to stronger representation of the relevant compared to irrelevant stimulus dimensions. No other main effects or interactions were significant (all *p*s > .11). One sample *t*-tests confirmed that these regions only encoded the task-relevant stimulus distinctions (mean accuracy (MA) 55.8%, [t(25) = 3.93, *p* < .001]) and not the irrelevant ones (MA 48.6%).

#### 3.2.2 Visual cortices

We tested whether information pertaining to task-relevant and task-irrelevant features was coded in BA17 (Fig. 3, right panel). An ANOVA with factors *relevancy* and *object* showed no main effects or interactions (all *p*s > .32). Thus, we found no evidence that context modulates coding of feature information in this region. However, BA 17 did show above chance classification of these objects according to both the relevant (relative to chance, MA 56.2%; [t(25) = 2.35, *p* = .002]) and the task-irrelevant (relative to chance; MA 55.8%; [t(25) = 2.95, *p* = .006]) stimulus features, as predicted for a stimulus-driven response.

We tested whether object-responsive cortex (LOC) coded task information using an ANOVA with factors *relevancy* and *object* (collapsed over left and right clusters). There were no main effects of interactions (all *p*s > .19). When we compared coding to chance, the LOC did not carry significant information about task relevant or irrelevant distinctions (all *p*s > .19).

#### 3.2.3 Inferior temporal cortex

As IT has previously been shown to be involved in categorical distinctions (e.g., Kriegeskorte et al., 2008) we tested whether this region coded information about the categorical distinctions in this paradigm (ANOVA with factors *relevancy* and *object*). There were no main effects or interactions (all *p*s > .1) or evidence of coding above chance for the relevant or irrelevant categorical distinctions of our novel objects (all *p*s > .1).

#### 3.2.4 Searchlight

To identify any additional regions coding task-relevant information, we conducted an exploratory analysis using a roaming searchlight. We assessed the results with cluster-level family wise error (FWE) correction for multiple comparisons (voxelwise threshold: *p* < .001 uncorrected). This revealed one large cluster, centred on the precuneus bilaterally and extending into the superior parietal lobe in both hemispheres [peak voxel at MNI co-ordinates (−10 −78 42), BA 7, cluster extent: 1526 voxels, FWE-corrected cluster-level *p* < .001]. At a more lenient cluster-level threshold (*p* < .05 uncorrected at the cluster level) coding of relevant object information was found in and around our MD ROIs in the left IFS [(−40 22 28), BA 44, cluster extent: 155, cluster-level *p* = .021], right IFS [(52 16 18), BA 44, cluster extent: 143, cluster-level *p* = .026], and at the boundary of the left IFA and AI/FO ROIs [(−32 28 8), BA 47, cluster extent: 169, cluster-level *p* = .017]. Three additional clusters were found in the cerebellum; [(−4 −80 −18), cluster extent: 223, cluster-level *p* = .007; (−36 −64 −30), cluster extent: 167, cluster-level *p* = .017; and (28−74 −22), cluster extent: 135, cluster-level *p* = .029]. A similar exploratory searchlight for irrelevant information coding revealed no significant clusters at either threshold.

For each voxel in the brain, we also performed a paired *t*-test to test for regions where relevant information was coded more strongly than irrelevant information. Again, one cluster survived FWE correction at the cluster-level (with a voxelwise threshold of *p* < .001) in the precuneus [(17−70 38), BA 7, cluster extent: 447, FWE-corrected cluster-level q < .001]. At an uncorrected threshold, clusters were found again in the MD system: IFS [(−28 40 16), BA 44, cluster extent: 166, cluster-level *p* = .015]; and two clusters in the ACC/pre-SMA [(6 20 50), BA 32, cluster extent: 144, cluster-level *p* = .023; and (8 36 18), BA 32, cluster extent: 106, cluster-level *p* = .045]. Additional regions were the frontal pole [(12 58 14), BA 10, cluster extent: 110, cluster-level *p* = .042] and the cerebellum [(−32 −66 −26), cluster extent: 177, cluster-level *p* = .013].

### 3.3 Coding of category placement

Given our paradigm, it was possible that as well as the categorisation decision at the level of the stimulus, participants also held a category *number* in mind on each trial. Therefore, we conducted an additional analysis in which the classifier was trained on the data representing the category number decisions in one task and tested on the category number decisions participants made in the other task context. The classifier did not successfully cross-classify category number placement of the objects in the MD system [mean classification accuracy 50.3%, t(25) = 1.2, *p* = .32]. We calculated the Bayes Factor using a default uniform prior (Love et al., 2015) to interpret this null effect (BF_10_ = .5). This approaches the level of .33 suggested by Jeffreys (1998) to represent strong evidence for the null hypothesis. Consistent with our previous work in a similar paradigm (Jackson et al., 2017), the evidence suggests that any MD activity patterns corresponding to category number did not generalise between the two tasks. This may be because the MD regions did not hold an abstract representation at the level of category number (e.g., “category 1” when it refers to “long” is encoded differently from “category 1” when it refers to “anti-clockwise”) or because our analysis did not capture an abstract representation that did in fact occur (e.g., a brief response later in the trial). For our purposes, however, this result suggests that any voxel re-use between codes in our main analysis (below) is unlikely to be driven by abstract representation of category number.

### 3.4 Voxel contribution to multiple neural codes

Our main analysis was an extension of multi-voxel pattern analysis which examined the extent to which the *same* MD voxels contributed to multiple codes for object information. We ran permutation tests to compare the proportion of voxel re-use we observed to that expected by chance. We carried out this analysis for all the ROIs that showed significant coding of the stimulus information.

Overall the MD network displayed a higher proportion of voxel re-use for the relevant dimensions than what would be expected by chance (23.9%, *p* < .01), suggesting that MD coding indeed reconfigures to solve different tasks. Considering these regions separately, voxel re-use was also above chance in the IPS (25.1%, *p* < .01) and IFS (27.9%, *p* < .05). Conversely, in BA 17 voxel re-use was not above chance for the relevant (*p* = .24) or the irrelevant information (*p* = .57).

We also examined voxel re-use in the additional regions, outside of the MD system, that the searchlight had found to represent relevant information. The precuneus cluster that survived FWE correction for coding of task-relevant information did not display a significant level of voxel re-use (*p* = .99). However, voxel re-use was above chance (28.5%, *p* = .002) in one of the cerebellar clusters [(−4 −80 −18)]. There was no evidence for above chance re-use in the other two cerebellar clusters (both *p*s = .99).

As a sanity check we tested whether voxel re-use was at chance when information coding was at chance, as in the case of irrelevant information coding in the MD system. Indeed, voxel re-use for irrelevant information was not different from chance across the MD ROIs (all ps > .12). Voxel re-use was also at chance in the LOC for relevant (*p* = .72) and irrelevant information (*p* = .85), and IT for relevant (*p* = .72) and irrelevant information (*p* = .81). Moreover, voxel re-use was significantly stronger for relevant relative to irrelevant information in the MD system (main effect of *relevancy* [F(1,25) = 4.52, *p* = .04], which did not interact with the factor *region* [F(3,78) = 2.37, *p* = .07], in a repeated measures ANOVA with factors *relevancy* and *region*.

## 4 Discussion

The MD network has been proposed to code information ‘adaptively’ (Duncan, 2010, 2013). The mechanism is described in terms of the responses of single neurons (Duncan, 2001), but previous work in humans has focused mainly on the response of whole brain regions (e.g., Jackson et al., 2017). To explore this mechanism in more detail we developed a method to measure the extent to which the *same voxels* in these regions contributed to coding of information across two distinct sets of objects and compared this to chance derived from permutation tests. We found that single MD voxels contributed to multiple codes for relevant object information, while voxels in the early visual cortex did not. Consistent with reports of single neuron flexibility in frontoparietal cortex of NHPs (e.g., Cromer et al., 2010), this finding emphasises the flexible response of the human MD regions.

The novel method in this study was developed to bridge the gap between region-level results in humans (e.g., Jackson et al., 2017) and detailed analysis at the single-unit level (e.g., Cromer et al., 2010). Our method allowed us to check whether individual voxels contributed signal to multiple neural codes. It is important to consider however, that voxel re-use is of course an indirect measure of the extent to which individual *neurons* are re-used. Even at this intermediate level of resolution it is possible that the key voxels which were re-used between codes happened to sample two independent populations of neurons each responding to the two different tasks. Ideally, to answer a question about whether neural resources are re-used across multiple tasks, we would exploit responses at the neural-level rather than the voxel-level. As this is not possible in these data, we draw conclusions only at a voxel level. However, it seems unlikely that such an explanation could completely account for these results, because the key independent neural populations (coding for the arbitrary categorisations) would have to happen to concentrate within single voxels more frequently than they are distributed across voxels, and this would have to be consistent across the MD regions and participants.

NHP studies have shown that higher cortical neurons adapt their tuning profiles to respond to information that is currently relevant (e.g., Freedman, 2001). In our previous work (Jackson et al., 2017) we showed that the human MD regions adjust their representations of single objects to emphasise task-relevant category distinctions, resulting in preferential coding of attended stimulus features. Here we show that these regions code the task-relevant category distinctions across distinct groups of objects, and, replicating our previous work, that coding of the attended features is stronger than coding of the irrelevant stimulus features. This stands in contrast to BA 17, which coded both relevant and irrelevant visual features, with no modulation by behavioural relevance. Consistent with the proposal that cognitive flexibility underlies MD involvement in a wide range of tasks (Cole & Schneider, 2007; Duncan & Owen, 2000; Duncan, 2010), these data emphasise that this system prioritises processing of the currently relevant features of a stimulus.

In our searchlight analysis, one additional region, centred on the precuneus, showed preferential coding of task-relevant information. Interestingly, this region, which is typically considered a major component of the default mode network (e.g., Cavanna, 2007; Fransson & Marrelec, 2008) and in turn associated with the task-negative or resting state (e.g., Fox et al., 2005; Raichle et al., 2001; Shulman et al., 1997), has recently been reported to hold representations relevant to active tasks (Crittenden, Mitchell, & Duncan, 2015; Woolgar, Afshar, et al., 2015). Here we found that the precuneus represented task-relevant object category information, which, similar to MD cortex, was stronger than its representation of irrelevant information. However, in the precuneus, unlike MD cortex, there was no evidence for flexible re-use of the neural resources of the precuneus (voxel re-use at chance) to achieve this representation.

How specific is voxel re-use to the MD system? Note that since re-use values will necessarily depend on the local vasculature and signal strength, they are only interpretable relative to the permutation test in the same region, so we did not perform direct comparisons of re-use values between ROIs. Therefore, our conclusion is limited to observing that reuse was above chance for the MD system and not for others. Of our *a priori* ROIs that coded object category, re-use was above chance in the MD system and not in BA17. Our other ROIs (LOC and IT) did not show coding of category, despite previous reports of categorical information in these regions with different stimuli and paradigms (e.g., Kriegeskorte et al., 2008; Mur et al., 2013). They therefore could not be expected to show re-use. However, our exploratory searchlight analysis revealed further regions that coded for task-relevant information: the precuneus and, at an uncorrected threshold, three clusters in the cerebellum. Of these, voxel re-use was above chance only in one of the cerebellar clusters. This suggests a degree of specificity, but also demonstrates that the MD system is not the *only* system in which voxels may be re-used. The cerebellar result may reflect the substantial cerebellar projections from the lateral prefrontal cortex (e.g., Ramnani et al., 2005) and its increasingly recognised role in executive functions (Bellebaum & Daum, 2007).

In this paper we refer to the extent to which multiple multivoxel patterns load heavily on the same sets of voxels as “voxel re-use”. In the NHP literature, the re-use of single neurons across multiple tasks has been called “multitasking” (Cromer et al., 2010), but we avoid this term to avoid confusion with the term multitasking in the human cognitive literature. Similar neural properties have also been described using the term “mixed selectivity” (Fusi, Miller, & Rigotti, 2016; Rigotti et al., 2013) whereby certain neural populations simultaneously reflect different task parameters. The emphasis in our work is slightly different in that we have examined the extent to which neural resources contribute to the representation of the same type of information (object information) in distinct categorisation tasks performed at different times. However, the concept of re-use could certainly also incorporate using the same resources in codes for different types of information.

As far as we are aware, this work constitutes the first to attempt to quantify voxel re-use in human data and accordingly it is difficult to predict what order of re-use values to expect. Average MD re-use amongst the top 10% of voxels was 23.9%. This is perhaps comparable to the NHP literature in which Roy et al. (2010) demonstrated that 24% of prefrontal neurons displayed this form of flexibility. However that group also reported an instance where a significantly higher proportion, 44% of prefrontal neurons, were re-used to code the relevant distinctions of two different tasks (Cromer et al., 2010). The authors suggested that the different extent of reuse between the studies depended on how physically different the two stimulus sets were: neural re-use was lower when stimuli were more similar to each other (and therefore, the task was more difficult). It would be interesting in the future to use the methods developed here to examine the extent to which voxel re-use varies with stimulus similarity and task demands.

Successful behaviour requires an adaptive cognitive system that can process information flexibly and efficiently. Our data suggests that the MD network demonstrates this type of flexibility, emphasising task-relevant features of different objects and flexibly re-using its resources to do so, providing a potential neural substrate for flexible behaviour. Future investigations can utilise the methods we describe here to consider the contribution of individual voxels alongside whole brain regions.

## Supplementary Material

Supplementary data related to this article can be found at https://doi.org/10.1016/j.cortex.2018.07.006.

Supplementary Methods
