# Supplementary material for: Adaptive coding in the human brain: Distinct object features are encoded by overlapping voxels in frontoparietal cortex
Source: Cortex. Author manuscript; Available in PMC 2019 Jul 15. (PMC6629547; doi:10.1016/j.cortex.2018.07.006)
Supplement: Supplementary Methods [file EMS83377-supplement-Supplementary_Methods.docx]

**Supplementary Methods

S1: Procedure for practice and titration**

Participants completed at least 6 blocks of practice trials. In these blocks stimuli were initially presented for 400ms until participants achieved >80% correct after which objects were presented for 216ms. Feedback (correct/incorrect) was presented after each response until participants achieved >80% performance, after which feedback (percent correct) was only given at the end of each block. Once participants reached >80% correct in both tasks, we titrated the stimuli to ensure no difference in reaction times between tasks. We matched the difficulty of the smoothy object task to the spiky object task by increasing or decreasing the difficulty of the smoothy task. We thresholded the difficulty of the smoothy task by switching smoothy object stimulus sets varying on the physical difference across the relevant decision boundary (e.g. larger physical difference in breadth dimension). This thresholding procedure was repeated until there was no difference in reaction time between the two task contexts, as assessed with Bayes analysis in each participant separately (BF < 1 taken as evidence for no difference between conditions).

Immediately prior to entering the scanner, participants completed a further 2 blocks of each task to avoid initial practice effects in the scanner. These blocks also introduced a response-mapping screen, which randomly assigned the button to be pressed for each category (clockwise or anti-clockwise spikes in the orientation task and narrow or wide spheroids in the breadth task) on a trial-by-trial basis. This allowed separate estimation of the blood-oxygen-level dependent (BOLD) response associated with the perceptual decision about each category from that associated with each button press. Participants also performed an additional two practice blocks in the scanner during the structural scan to familiarise them with the button-response box in the scanner.

**S2: Steps for decoding of task information.**In total, there were 16 blocks for each participant in each experiment: 8 with orientation (spiky) relevant and 8 with breadth (smoothy) relevant. For each classification, we used a leave-one-out 8 fold splitter whereby the classifier was trained using the data from 7 out of the 8 blocks and subsequently tested on its accuracy at classifying the unseen data from the remaining block, iterating over all possible combinations of training and testing blocks. The accuracies were then averaged to give a mean accuracy score. This procedure was repeated for each condition, participant and ROI separately.

The mean classification accuracy for each participant in each ROI and in each condition, was then entered into a second level analysis. We conducted a three factor analysis of variance (ANOVA) on classifier accuracy with the factors *relevancy* (Task-relevant, Task-irrelevant, *object* (Spiky, Smoothy), and *MD region* (AI/FO, IFS, ACC/pre-SMA, and the IPS; collapsed across hemisphere where appropriate)*.* Since a difference in coding in the relevant and irrelevant conditions is only interpretable if coding in at least one condition is also significantly above chance, we also conducted one-sample t-tests against the classification accuracy expected by chance (50%) in each condition (relevant and irrelevant) separately. One-tailed significance tests were used where appropriate for inference: tests comparing classification accuracy to chance are one-tailed as below chance classifications are not interpretable. All other tests are two-tailed. Alpha was adjusted for four comparisons using Bonferroni correction (0.05 divided by 4).

We also examined whether coding in the visual cortices and IT was stronger for task-relevant than irrelevant stimulus features. For this, we used a two-factor ANOVA on classifier accuracy with factors *relevancy* and *object* (BA17/LOC/IT) collapsed across hemisphere. Again, we also tested whether coding in the visual cortices/IT was above chance in each condition separately (one-sample t-tests).

**S3: Permutation test.**We conducted the following test for each region separately. In the first step, we exhaustively permuted the condition labels within each block (128 combinations total) for each person and each task separately. For each permutation, we trained a classifier using the permuted data, and calculated the transformed weight vectors and voxel re-use index, in the same way as we had done for the correctly labelled data. Next, we built a group level null distribution by sampling (with replacement) from the set of 20 participants * 128 permutation results (one sample per participant per permutation, 10,000 permutations). From this, we calculated the probability *p* of observing the actual voxel re-use value (from the correctly labelled data) given the group null distribution, using the Monte-Carlo approach (*p* = *k*+1/(*n*+1) where *k* is the number of permutations in the null with equal or higher accuracy to the actual voxel re-use value and *n* is the number of all permutations.
